# Energy Drinks: A Reversible Risk Factor for Atrophic Gastritis and Gastric Intestinal Metaplasia

**DOI:** 10.7759/cureus.12298

**Published:** 2020-12-26

**Authors:** Anisha Garg, Andrea Rodriguez, Jason T Lewis, Rishabh Bansal, Bhaumik Brahmbhatt

**Affiliations:** 1 Gastroenterology, Mayo Clinic, Jacksonville, USA; 2 Gastroenterology, Mount Sinai Medical Center, Miami, USA; 3 Pathology, Mayo Clinic, Jacksonville, USA

**Keywords:** energy drinks, atrophic gastritis, gastric intestinal metaplasia, gastric cancer

## Abstract

Energy drinks (ED) are becoming increasingly popular, but little has been reported about their stomach effects. To our knowledge, there is no literature suggesting an association with the development of atrophic gastritis (AG) or gastric intestinal metaplasia (GIM). AG and GIM have been associated with an increased risk of gastric cancer. Reversal of these lesions has shown to reduce the incidence of gastric cancer but has only been studied to eradicate Helicobacter pylori. This case describes a female who consumed high amounts of ED and was subsequently diagnosed with AG and GIM. Interestingly, the pathologies resolved upon cessation of ED.

## Introduction

Gastric cancer is the fifth most common cancer globally and the third leading cause of cancer-related mortality. Atrophic gastritis (AG) and Gastric intestinal metaplasia (GIM) have an increased risk of developing into gastric cancer by following the gastric carcinogenesis pathways [1]. Reversal of AG and GIM has shown to reduce the incidence of gastric cancer. However, it has only been studied to eradicate the most common cause, Helicobacter pylori (H. pylori) [2-3]. We present AG and GIM's case involving a 34-year-old Hispanic female regularly consuming energy drinks (ED) since 2003 with subsequent reversal of AG and GIM upon cessation of ED. This is the first case report describing ED as a possible risk factor for AG and GIM development.

## Case presentation

**Figure 1:**
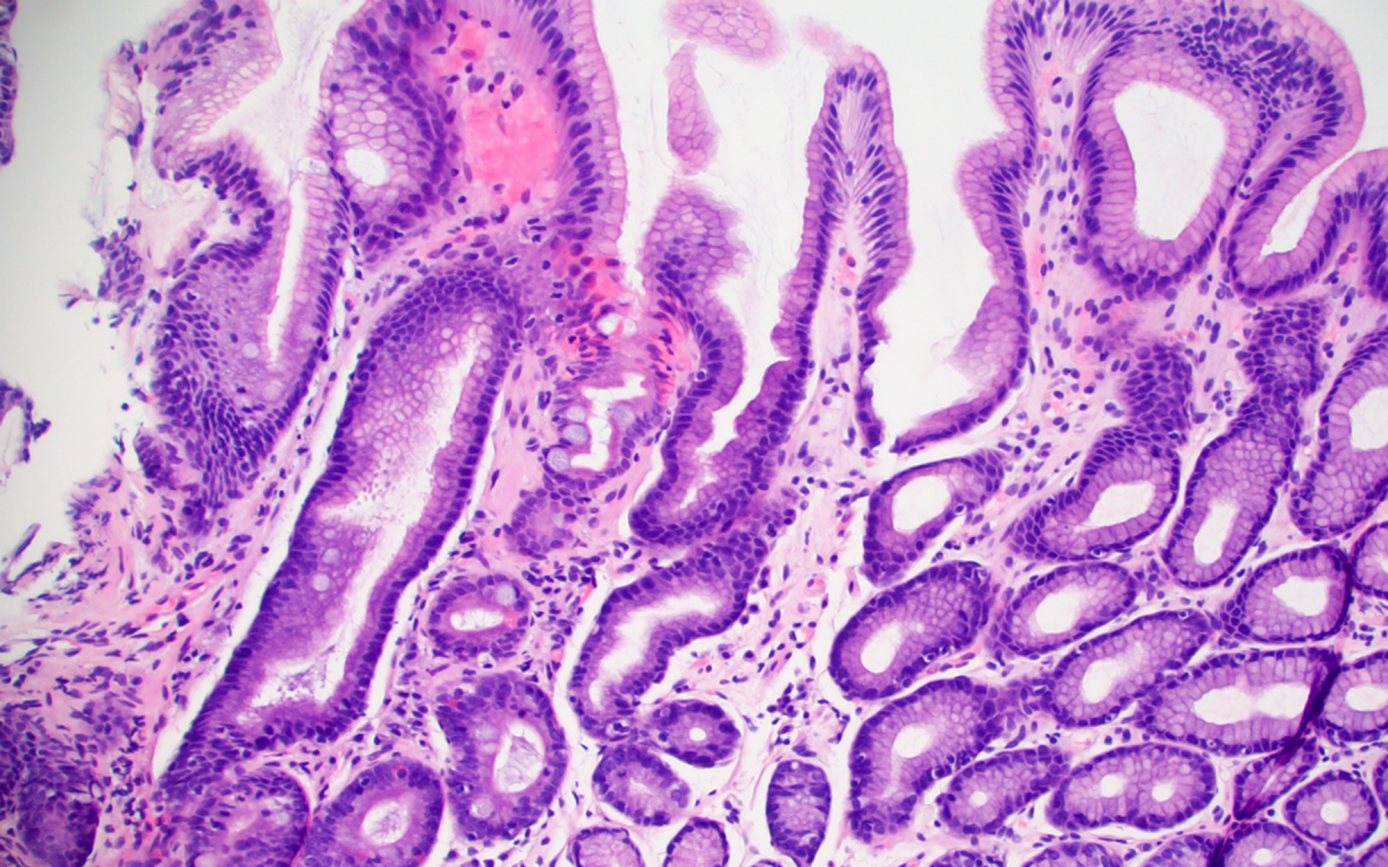
Antral mucosa with changes of reactive gastropathy and atrophy. Intestinal metaplasia, characterized by goblet cells, is seen within the mid-left portion of the image.

**Figure 2:**
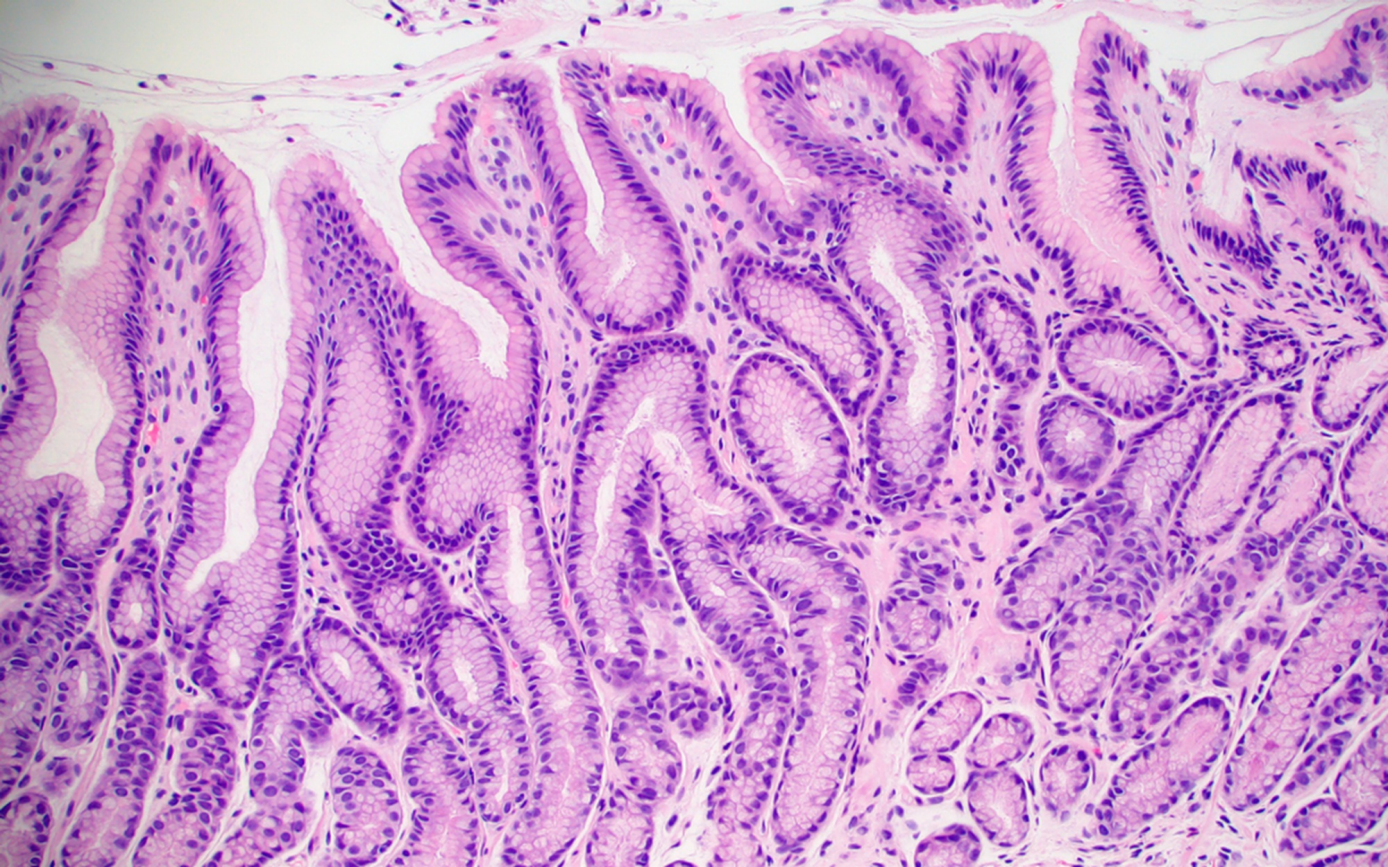
Normal antral mucosa. No reactive changes or intestinal metaplasia present.

A 34-year-old Hispanic female presented with two different types of abdomen pain for more than a year. The first pain was in the epigastric area and was associated with dyspepsia. The second one was in the right upper quadrant and was colicky. No abnormality of the gallbladder or the biliary tract was found when the patient underwent abdominal ultrasonography and contrast-enhanced magnetic resonance cholangiopancreatography (MRCP). On an esophagogastroduodenoscopy (EGD), the stomach appeared atrophic raising suspicion of intestinal metaplasia. Biopsies taken from the stomach revealed AG and focal antral intestinal metaplasia (Figure 1). H. pylori immunostaining was negative. The patient had normal levels of iron, vitamin B12, methylmalonic acid, gastrin, intrinsic factor blocking antibody, and parietal cell antibody. The patient reportedly consumed alcohol occasionally a few times per year and had never smoked in her lifetime. On further questioning, she reported a regular intake of 1-2 ED/day, namely Red Bull® and Monster Energy® for the past 15 years. The patient was hence advised to stop consuming ED, and her dyspepsia was treated with intermittent proton pump inhibitors for the next two years. Surveillance EGD 2 years after the initial diagnosis revealed normal-appearing gastric mucosa. Gastric topographic mapping biopsies using Sydney protocol revealed normal mucosa (Figure 2). These findings implicated the reversal of AG and GIM upon withdrawal of ED.

## Discussion

In the study conducted by Sonnenberg A et al. using biopsies from upper endoscopies between 2008 and 2013, the overall prevalence of GIM was 4.9% [4]. Since chronic AG is frequently asymptomatic, its prevalence is not known [5] Chronic H. pylori infection is the most common etiologic factor identified in the development of AG and GIM, followed by Autoimmune Metaplastic Atrophic Gastritis (AMAG). The worldwide prevalence of H. pylori is approximately 50%, and approximately 35%-40% in the United States [6]. In a study done by Shiota S et al. at VA Medical Center, it was found that out of all patients with gastritis, approximately 18% of the patients had H. pylori-negative gastritis [7].

On the other hand, the prevalence of AMAG is only 2% in the general population [8]. Therefore, the prevalence of H. pylori-negative gastritis far outnumbers AMAG, making us think that there must be other causes than the two established ones. According to recent studies, factors like smoking and diet play an important role in AG's pathogenesis. Smoking has been shown to promote AG and GIM, but the relationship has been established only in H. pylori-positive patients [9]. High salt intake has also shown to be associated with increased risk of AG [10].

On the other hand, moderate alcohol intake has been inversely associated with AG suggested by the facilitation of elimination of H. pylori through the antibacterial action of alcohol [11]. Our patient has never smoked in her lifetime and consumes a low salt diet, ruling out the role of smoking and high salt diet in the development of her gastric pathologies. However, her long history of regular consumption of ED suggests that these could be another dietary factor involved in AG and GIM's pathogenesis.

A study performed to examine the effects of Power Horse® ED on Albino rats' stomach, and pancreas showed evidence of Power Horse-induced oxidant-antioxidant imbalance, degenerative changes, and increased apoptosis in the fundic mucosa of the stomach of the rats [12]. The fundic mucosa showed reduced parietal cells and gastrin hormone expression compared to the control group [12]. Another study done by Raeesa A. Mohamed et al. on adult Wistar rats demonstrated marked depletion of mucus secretion in the mucosae of stomach and duodenum of Red Bull group with a significant increase of apoptotic cells in the mucosae of stomach and duodenum [13]. Unfortunately, no data is describing the effects of ED on the mucosa of the human stomach. The only gastrointestinal (GI) adverse effects reported in humans include nausea, vomiting and diarrhoea [14].

All ED share ingredients constitute caffeine, carbonated water, glucose, sucrose, citric acid, sodium citrate, taurine, B vitamins (B2, B3, B5, B6, B12) natural or artificial flavours. Power Horse also contains inositol and glucuronolactone, both found in Monster ED but not in Red Bull. Red bull differs from Power horse as it contains acidity regulators such as Sodium Bicarbonate and magnesium carbonate. On the other hand, Monster ED differs from Power horse as the former contains additional amino acids (L-carnitine), preservatives (sorbic acid, benzoic acid), plant extracts (guarana seed extract, Panax ginseng root extract) and sucralose. GI side effects described with caffeine include increased gastric acid production, gastro-oesophagal reflux, decreased absorption and impaired motility [15], but data regarding these are conflicting and inconclusive. Several studies have also shown that these side effects might be due to the other ingredients present in caffeine-containing drinks rather than caffeine itself [16]. Sodium Citrate ingestion has been shown to cause nausea, bloating, vomiting and diarrhoea [17]. The rest of the ingredients, individually, have not been reported to cause any GI adverse effects. None of the ingredients, however, have been associated with the development of AG or GIM.

The reversibility of AG and GIM, till now, has been documented only with the eradication of H. pylori [3]. Substances like muscovite, folic acid and gefarnate may also be useful in reversing AG and GIM [18-20]. Nonetheless, there is no known literature suggesting reversibility of the lesions with the cessation of energy drinks.

## Conclusions

Energy drink discontinuation in our patient led to the reversal of her gastric pathology showing environmental factors could play a critical role in gastric dysplasia. Prompt recognition and lifestyle modification may prevent AG, GIM, and subsequently gastric cancer. Hence we need more data about the effects of ED to know for possible reversal of AG and GIM to minimize symptoms, surveillance, and risk of gastric cancer. Additional research is also warranted on what particular ingredient(s) in ED, at what concentration, and what duration, could lead to the development of the observed findings.

## References

[1] Kapadia CR (2003). Gastric atrophy, metaplasia, and dysplasia: a clinical perspective. J Clin Gastroenterol.

[2] Lee YC, Chiang TH, Chou CK, Tu YK, Liao WC, Wu MS, Graham DY (2016). Association between Helicobacter pylori eradication and gastric cancer incidence: a systematic review and meta-analysis. Gastroenterology.

[3] Hwang YJ, Kim N, Lee HS (2018). Reversibility of atrophic gastritis and intestinal metaplasia after Helicobacter pylori eradication - a prospective study for up to 10 years. Aliment Pharmacol Ther.

[4] Sonnenberg A, Genta RM (2015). Changes in the gastric mucosa with aging. Clin Gastroenterol Hepatol.

[5] Rodriguez-Castro KI, Franceschi M, Noto A (2018). Clinical manifestations of chronic atrophic gastritis. Acta Biomed.

[6] Lacy BE, Rosemore J (2001). Helicobacter pylori: ulcers and more: the beginning of an era. J Nutr.

[7] Shiota S, Thrift AP, Green L (2017). Clinical manifestations of Helicobacter pylori-negative gastritis. Clin Gastroenterol Hepatol.

[8] Jacobson DL, Gange SJ, Rose NR, Graham NM (1997). Epidemiology and estimated population burden of selected autoimmune diseases in the United States. Clin Immunol Immunopathol.

[9] Nakamura M, Haruma K, Kamada T (2002). Cigarette smoking promotes atrophic gastritis in Helicobacter pylori-positive subjects. Dig Dis Sci.

[10] Song JH, Kim YS, Heo NJ (2017). High salt intake is associated with atrophic gastritis with intestinal metaplasia. Cancer Epidemiol Biomarkers Prev.

[11] Gao L, Weck MN, Stegmaier C, Rothenbacher D, Brenner H (2009). Alcohol consumption and chronic atrophic gastritis: population-based study among 9,444 older adults from Germany. Int J Cancer.

[12] Ayuob N, ElBeshbeishy R (2016). Impact of an energy drink on the structure of stomach and pancreas of albino rat: can omega-3 provide a protection?. PLoS One.

[13] Mohamed RA, Ahmed AM, Al-Matrafi TA (2018). Energy drinks induce adverse histopathological changes in gastric and duodenal mucosae of rats. Int J Advances Appl Sci.

[14] Hammond D, Reid JL, Zukowski S (2018). Adverse effects of caffeinated energy drinks among youth and young adults in Canada: a web-based survey. CMAJ Open.

[15] Shearer J (2014). Methodological and metabolic considerations in the study of caffeine-containing energy drinks. Nutrition Reviews.

[16] Börger HW, Schafmayer A, Arnold R, Becker HD, Creutzfeldt W (1976). The influence of coffee and caffeine on gastrin and acid secretion in man [Article in German]. Dtsch Med Wochenschr.

[17] Urwin CS, Dwyer DB, Carr AJ (2016). Induced alkalosis and gastrointestinal symptoms after sodium citrate ingestion: a dose-response investigation. Int J Sport Nutr Exerc Metab.

[18] Wang LJ, Zhou QY, Chen Y (2009). Muscovite reverses gastric gland atrophy and intestinal metaplasia by promoting cell proliferation in rats with atrophic gastritis. Digestion.

[19] Zhu S, Mason J, Shi Y (2003). The effect of folic acid on the development of stomach and other gastrointestinal cancers. Chin Med J (Engl).

[20] Lai Y, Xu P, Li Q, Ren D, Sun X, Xu K, Huang J (2013). Evaluation of efficacy of gefarnate in treatment of chronic atrophic gastritis with intestinal metaplasia. Chinese J Gastroenterol.

